# Benzoate of Soda in Diphtheria

**Published:** 1880-04

**Authors:** 


### BENZOATE OF SODA IN DIPHTHERIA.

Dr. Letzerich has successfully treated, with benzoate of soda, 27 cases of diphtheria which came under his care during an epidemic of the disease in Berlin. Of these cases eight were severe, accompanied by high fever, delirium, retention of the urine and feces, existing often before the extensive local affection had made its appearance. In the blood there was found numerous bacteria and plasma corpuscles, from which by cultivation in veal broth, very large colonies of micrococci became developed. The dose of sodium benzoate for children and adults is to be regulated by the weight of the body. The formula for infants under one year old is

℞

| | |
|---|---|
| Soda Benzoate. pur. . . . | 5.0 |
| Aquæ Destillat. . . . . | |
| Aquæ Menth. pip., *aa* . . . | 40.0 |
| Syrup, Cort. Aurant. . . . . | 10.0 |

Half teaspoonful every hour.

The dose for children between one and three years of age is given at 7—8 grams in the course of a day; for children between three and seven years, 8—10 grams. Over seven years old, 10—15 grams, to be taken daily; no unpleasant effects have been observed even in young infants. The diphtheric membrane was sprinkled with the benzoate of soda in powder applied through a glass tube or quill. There is no slough formed, and thereby the danger is averted of its acting as a firm covering under which an energetic development and growth of the organism can take place.

The insufflation was made every three hours in severe cases; in the mild forms two or three times daily. The author also recommends this remedy in gastric or intestinal catarrh, particularly of infants, and states that at times the results are surprising in these latter cases. He firmly believes in the statement of Klebs, that it is to be recommended in all diseases which originate by infection.—*Boston Med. and Sur. Jour., from Berlin Klein. Wochs.*

